# Neuroendocrine tumors in Panama: A nationwide database analysis

**DOI:** 10.3892/mco.2021.2319

**Published:** 2021-06-11

**Authors:** Moises Cukier, Ruth Vergara, Jorge D. Mendez-Rios, Omar Castillo, Irma Barrera, Eliecer Tello, Olivia El Achtar, Yong Loo, Hector Tapia, Guadalupe Perez, Maximino Peña

**Affiliations:** 1 Department of Surgical Oncology, Instituto Oncologico Nacional, Panama City, Panama 0816, Republic of Panama; 2 Department of Pathology, Instituto Oncologico Nacional, Panama City, Panama 0816, Republic of Panama; 3 Department of Genetics and Cytogenetics, Caja de Seguro Social, Panama City, Panama 0824, Republic of Panama; 4 Department of Genetics, School of Medicine, Universidad Interamericana de Panama, Panama City, Panama 0820, Republic of Panama; 5 Department of Medical Oncology, Instituto Oncologico Nacional, Panama City, Panama 0816, Republic of Panama; 6 Department of General Surgery, Caja de Seguro Social, Panama City, Panama 0824, Republic of Panama; 7 Department of Radiology and Interventionism, Instituto Oncologico Nacional, Panama City, Panama 0816, Republic of Panama; 8 Department of Endocrinology, Caja de Seguro Social, Panama City, Panama 0824, Republic of Panama; 9 Department of Gastroenterology, Instituto Oncologico Nacional, Panama City, Panama 0816, Republic of Panama

**Keywords:** neuroendocrine tumors, epidemiology, clinical data, Republic of Panama

## Abstract

Neuroendocrine tumors (NETs) comprise a heterogenous group of rare malignancies, which are increasing in incidence worldwide. To further understand the epidemiology of NETs in the Republic of Panama, the present study used two study groups, which included patients from several hospitals and clinics throughout the country, who were referred to the three largest national reference centers: The Complejo Hospitalario Metropolitano, Hospital Santo Tomas and Instituto Oncologico Nacional. These two groups comprised a retrospective cohort, which included cases reported between 2016 and 2017, and a second cohort, which was retrospective, but data were continuously collected from patients diagnosed with NETs between 2018 and 2019. Data from 157 patients with NETs reported that 83% of patients were in the 40-80 years old age group. The majority of cases (46%) presented as grade G1 tumors, while 29% were G3. Computerized tomography scans with contrast, and analysis of the Ki-67 biomarker and immunohistology markers (chromogranin A and synaptophysin) was performed in the majority of the cases. The results revealed that the most frequent anatomical sites for the primary tumor were the colorectum (17.2%), pancreas (12.7%) and stomach (12.1%), and the most frequent organ with metastasis was the liver, accounting for 34% of all cases. In conclusion, the present study is the first comprehensive study of NET in Panama to the best of our knowledge, which provides evidence of the demographic characteristics of the population, clinical features and overall survival for the affected population in this Central American country.

## Introduction

Neuroendocrine tumors (NETs) comprise a heterogeneous group of solid malignancies that frequently occur in the intestine, but have also been found to originate from other organs. The incidence of NETs is increasing worldwide; however, the associated cause remains unknown (1-5). The clinical manifestations of NETs vary widely depending on the location of the tumor and, in some cases, the type of hormones secreted, which promotes non-specific symptoms, such as diarrhea, bronchospasms, flushing, cardiac valve disease and other non-specific debilitating symptoms (6). When the NETs secrete hormones (functional tumors), clinical manifestations are common (4). Patients who present with a functional NET may complain of abdominal discomfort, which may be mistaken for irritable bowel syndrome, or other non-specific symptoms that can lead to misdiagnosis of dyspepsia and other benign conditions (6). When the NET is non-functional, the patient may be asymptomatic and the tumor is frequently left undetected. This causes a delay in diagnosis and a worse prognosis (7).

Research groups in Latin America have described the epidemiology of NET cases in their countries. One group from Argentina published a study in 2014 in which 532 cases were included to describe the epidemiology, prevalence, demography, symptoms and diagnostic methods for NETs (8). A similar study was conducted in Chile (9) published in 2019, that consisted in 166 patients with NETs. Interestingly, the demographic data from these two studies showed that patient age at diagnosis and sex were similar, but with a predominance of small-bowel NETs in the Chilean cohort. To the best of our knowledge, there have been no comprehensive studies firmly establishing the incidence, clinical characteristics, diagnostics procedures used or overall survival for NET cases in Panama to date. The present study represents the first comprehensive effort to determine the epidemiological characteristics, associated epidemiological factors and survival of patients with NETs in the Republic of Panama. Through a collaborative nationwide network that included surgical and medical oncologists, interventional radiologists and radio-oncologists, the present study was undertaken to establish a Panamanian NET database with the aim of improving our understanding of this rare condition.

## Patients and methods

### 

#### Study design

Both retrospective studies were conducted using the hospital medical records of patients from all over the Republic of Panama who were referred to the three largest national referral hospitals: The Complejo Hospitalario Metropolitano (CHM), Hospital Santo Tomas (HST) and Instituto Oncologico Nacional (ION). All patients, regardless of the age, with complete diagnosis record of NET were included. Patients with unconfirmed diagnosis by histopathology were not included. All three hospitals are located near the country's capital, Panama City. The study proposal was submitted to the Institutional Ethics Board Committee at the ION (Panama City, Panama), and was granted both national and institutional approval. The retrospective part of the study included 64 patients, who were treated between January 2016 and December 2017. The second group comprised 93 patients, for whom data were collected continuously between January 2018 and December 2019. A total of 157 cases were included in both cohorts. Data collection was concluded in January 2020. Cases were evaluated by a multidisciplinary team including surgical oncology, medical oncology, interventional radiology and radiation oncology specialists. Treatment was conducted based on international guidelines; details are not included, since this was outside the scope of the present study.

Histopathological diagnosis, classification and grading of NETs were determined based on paraffin-embedded block results reported for tissues analyzed at ION, which included biopsies and/or resection samples. The nomenclature and classification used for these tumors was based on the 2010 guidelines of the World Health Organization (10). The analysis of the levels of the biomarkers CD56, chromogranin A (CgA), synaptophysin (SYN) and Ki-67 were performed using immunohistochemistry and tumor behavior assessment. Fixation and staining of samples had been conducted using institutional protocols that included 4-µm sections and immunohistochemistry assays using anti-SYN (cat. no. MRQ-40; Merck KGaA), anti-CgA (cat. no. LK2H10; Ventana Medical Systems, Inc.; Roche Diagnostics), CD-56 (cat. no. 123C3; Dako; Agilent Technologies, Inc.) and anti-human Ki-67 (cat. no. M7240; clone MIB-1; Dako, Agilent Technologies, Inc.) antibodies, and did not constitute part of the present study. The collected data were curated and anonymized prior to analysis by removing all personal identifiable information.

Data for both arms of the study were independently collected. The data from the two groups had similar variables, and were combined in a single Excel spreadsheet, followed by manual curation of entries to confirm consistency, elimination of duplicate records and anonymization of the data. The final data were compiled into a database with 157 unique identifiers. Cases from the retrospective arm of the study are referred to as cohort 1, whereas cases from the second group, for which data were continuously collected, were identified as cohort 2. The unified database contained information on the epidemiology, diagnosis, treatment, levels of tumor markers and patient age at diagnosis.

#### Statistical analysis

Statistical analysis was performed using EpiInfo v7.2.4.0 software (Centers for Disease Control) and a 95% confidence interval (CI) estimation. SPSS v23.0 software (IBM Corp.) was used to perform descriptive univariate analysis of the frequencies and to determine the statistical significance of the geographical distribution of cases (11,12). For the Kaplan-Meier survival curves, data were stratified by age groups as follows: <40, 40-59, 60-79 and ≥80 years. Mantel Cox tests were applied to detect statistically significant differences between groups. The overall survival (OS) by tumor grade and anatomical site of the primary tumor was also determined. For variables presented on all tables, 95% CIs were determined. P<0.05 was considered to indicate a statistically significant difference.

## Results

### 

#### Age and sex distribution of patients with NETs

Data from a total of 157 NET cases were collected from the three main tertiary referral hospitals in the Republic of Panama. The age was similar between cohort 1 (mean, 61 years; range, 21-93 years) and cohort 2 (mean, 59 years; range, 20-90 years) (Table I). Male:female sex distribution was different between the two groups (1:1, respectively, in cohort 1; and 1:2, respectively, in cohort 2). Since the Republic of Panama has a population of ~4.14 million, the incidence of NETs in the country between 2016 and 2019 was calculated to be 0.92 cases for every 100,000 individuals, using National Registry Census data for those years (13).

#### Age and tumor grade of patients with NETs

The mean age of patients with NETs was 60 years, and the majority of the cases were identified in the 60-80 years (46.50%) and 40-59 years (36.94%) age groups. In total, <17% of all cases were observed in patients aged <40 or >80 years. A total of 72 patients (45.86%) also exhibited metastasis. The majority of the tumors (46.50%) were classified as grade G1, followed by G3 (28.66%) and G2 (19.75%). In 5.10% of the cases, tumor grade was undetermined or not available. Tumor grading information for each primary tumor location is presented in Table SI.

#### Geographical distribution of patients with NETs

**Figure 1:**
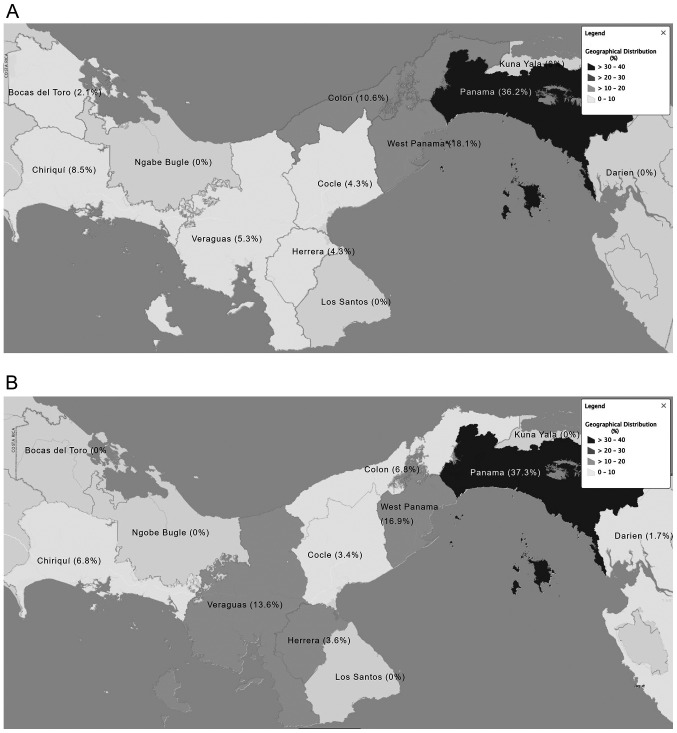
Frequency of neuroendocrine tumor cases by region. Frequency of cases depicted as a heat map throughout the provinces of Panama for (A) cohort 1 and (B) cohort 2. Dark gray, higher percentage of cases; light gray, lower percentage of cases.

The geographical distribution of cases before referral throughout the Republic of Panama is presented in Fig. 1. In total, 37.3% of all cases were found to have originated from the Panama province, 16.9% from the West Panama province and 13.6% originated from the Veraguas province.

#### Diagnosis, staging, classification and tumor grading of patients with NETs

The diagnosis, staging, classification and tumor grading of patients with NETs were performed using non-invasive and invasive procedures, imaging techniques and biomarker expression levels (Table II). The most common imaging modality used was contrast-enhanced CT scans, in which contrast was reported in 59.38% of cases. Colonoscopies and endoscopies were performed in 13.28 and 10.94% of cases, respectively. In addition, 9.38% of the patients underwent an ultrasound examination. Other diagnostic methods, such as mammography, radiography, magnetic resonance imaging, bone scan, cystoscopy and colposcopy accounted for 7.02% of all cases included in both cohorts.

The expression levels of markers, including Ki-67, CgA, SYN and CD56, were analyzed in the majority of cases in both study groups for grading and diagnostic purposes. The test yield is shown in Table III, but this did not include cases with undetermined or unavailable results. SYN was the most frequent marker used for diagnostic purposes (109 cases) and provided a test yield of 67.52%. In addition, CgA was used for cytological evaluation in 104 cases, demonstrating a test yield of 55.41%; none of the patients were using proton-pump inhibitors at the time of measuring CgA levels. The % of Ki-67 staining was used for tumor grading and prognostic purposes, providing a test yield of 52.20%. The expression levels of the less frequently used marker, CD56, were analyzed in 31 cases and showed a test yield of 19.11%.

#### Anatomical location of primary and metastatic NETs

The anatomical location of the primary NET was determined and the frequency of lesions at each location is summarized in Table IV. The data revealed that the three most frequent locations of the primary tumor were the colorectum (17.20% of cases), pancreas (12.74% of cases) and stomach (12.10% of cases) in the combined cohort. In 7.0% of cases, the location of the primary tumor was unknown or undetermined.

The number of cases for each metastatic location was also determined (Table V). The liver was the most frequent metastatic site in both study groups (data not shown), as 53 cases (33.76%) presented with hepatic metastasis. Within this group, patients with liver metastasis also had other affected organs, such as the peritoneum in 4.46% of cases and the lungs in 2.55% of cases. The brain followed as the second most common single metastatic site, with 2.55% of cases, followed by bone and peritoneal metastases in 1.27% of cases for each. Overall, other organs were identified as metastatic sites (7.01%) but these occurred less frequently. Metastasis was not determined or specified in 54.14% of cases.

#### Survival analysis of patients with NETs

The patients were treated according to the degree of tumor differentiation. Patients with differentiated tumors mainly received treatment with the somatostatin analogue, octreotide, and second-line treatment with targeted therapies, such as mTOR inhibitors, regardless of whether the tumors were functional or non-functional, due to the lack of functional imaging facilities in the country (data not shown).

**Figure 2:**
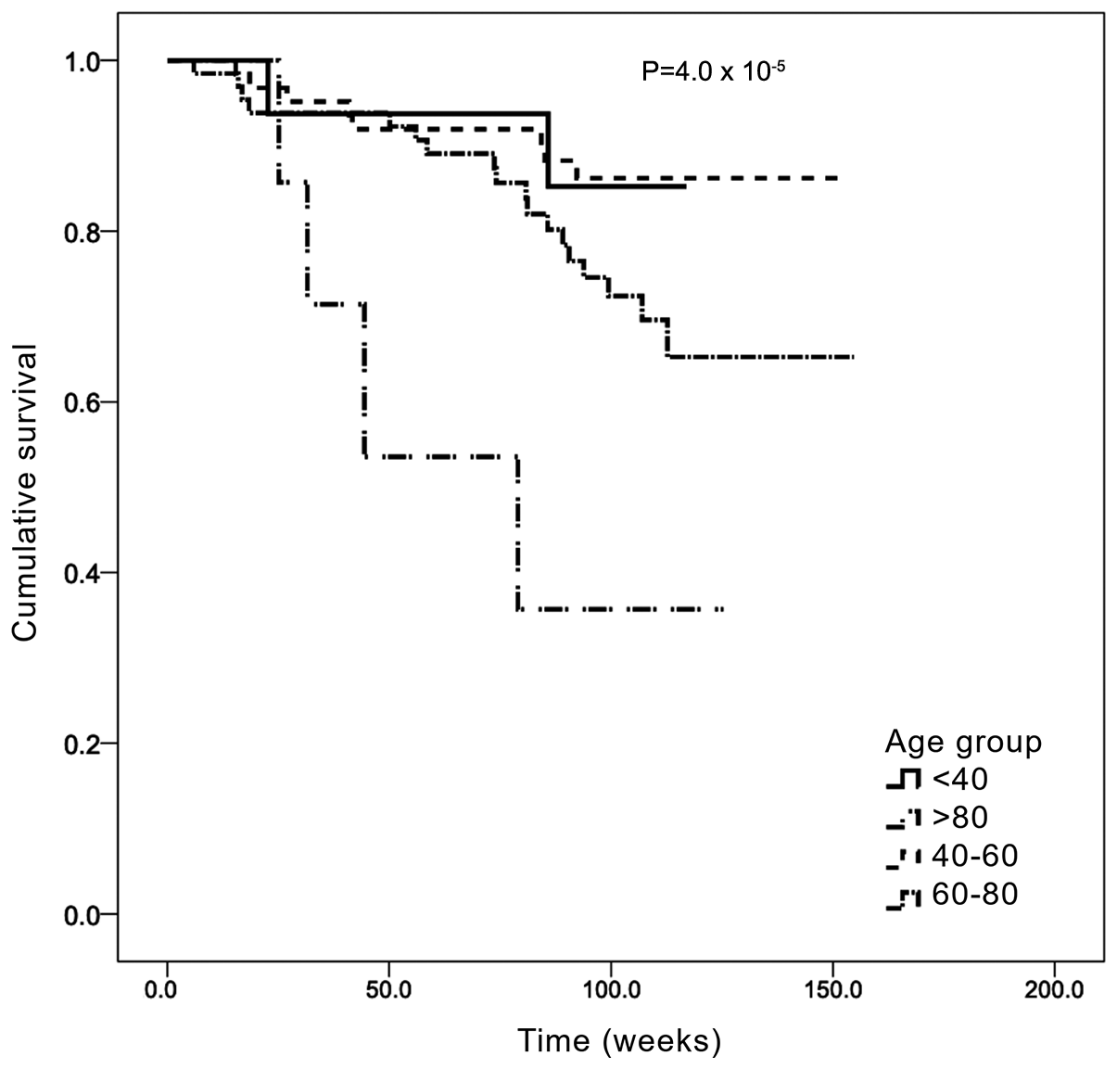
Kaplan-Meier analysis of overall survival by age group. Age groups were defined as follows: <40, 40-59, 60-79 and ≥80 years. The x axis represents the accumulative survival as a fraction (cumulative survival) and the y axis represents the survival time in weeks.

Survival analysis was first stratified by age group (Fig. 2). The median OS time was 132.3 weeks (95% CI: 125.2-139.1) and the median follow-up time was 100 weeks (~2 years). The 40-59 years age group had the longest OS, with a mean OS of 139.4 weeks (95% CI: 130.2-148.5), while the >80 years age group had the shortest OS, with a mean of 68.8 weeks (95% CI: 41.4-96.2). The differences between these groups were statistically significant (Mantel Cox χ^2^ =23.0; P=0.00004).

**Figure 3:**
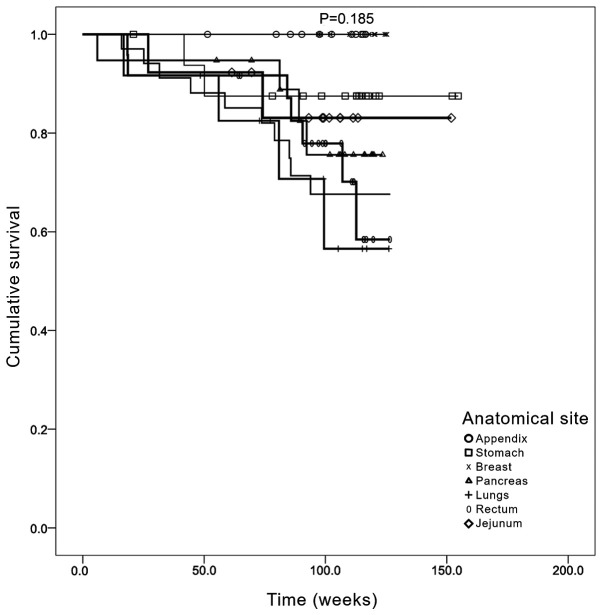
Kaplan-Meier analysis of overall survival by tumor grade. The x axis represents the accumulative survival as a fraction (cumulative survival) and the y axis represents the survival time in weeks.

The OS according to the tumor grade is presented in Fig. 3. Study subjects had a mean survival of 132.2 weeks (95% CI: 124.8-139.5). Patients in the G1 group had the longest OS, with a mean OS of 142.9 weeks (95% CI: 135.1-150.8), while patients in the G3 group had the shortest OS survival, with a mean OS of 96.8 weeks (95% CI: 84.0-109.7). The differences between these groups were also found to be statistically significant (Mantel Cox χ^2^=10.755; P=0.005).

**Figure 4:**
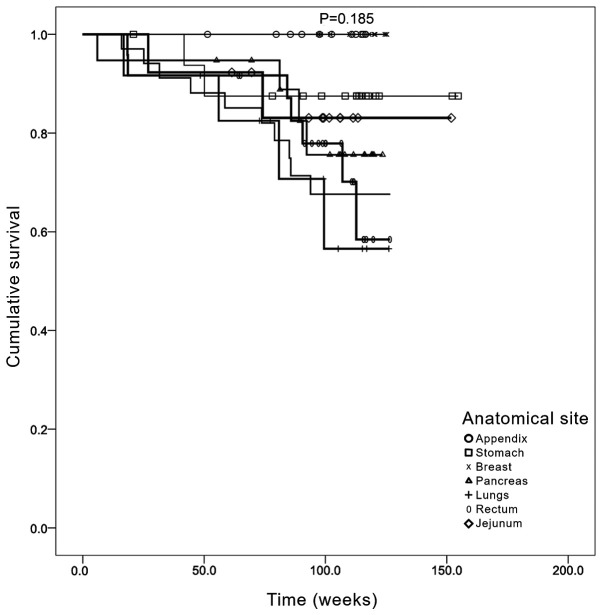
Kaplan-Meier analysis of overall survival by anatomical site of primary tumor. The x axis represents the accumulative survival as a fraction (cumulative survival) and the y axis represents the survival time in weeks.

OS was subsequently determined according to the anatomical site of the primary tumor (Fig. 4). The data revealed that those cases in which the primary tumor was located in the lung had the worse OS. This was followed by cases that had tumors localized in the colorectal region and pancreas. However, no statistically significant differences were identified between the different primary locations when stratified by treatment (Mantel Cox χ^2^=3.27; P=0.185).

## Discussion

NETs comprise a group of rare neoplasms that frequently affect the gastrointestinal and bronchopulmonary tissues (5); however, they can also develop in other tissues and organs, including the breast, ovaries and skin. Since NETs are of endocrine and neurological origin, they may secrete hormones that are associated with specific symptoms (6,7). In the earliest stages of the disease, patients with NETs may by asymptomatic or have non-functional tumors (14); thus, the absence of signs of disease may delay diagnosis (6). For this reason, it is paramount to understand the epidemiology of NET cases and raise awareness of this disease in Panama. To the best of our knowledge, the present study was the first comprehensive nationwide study in the Republic of Panama to characterize patients with NETs in two cohorts, between 2016 and 2017 and between 2018 and 2019.

The Panama and West Panama provinces are located in close proximity to each other and have the largest populations in the country (15). In the current study, these two provinces received all suspected cases from the other provinces, which partially explains the higher number of cases in these provinces. For example, it was reported herein that Panama province had 37.3% of all NET cases, while West Panama province had 16.9%. The largest tertiary referral hospitals are located in these two provinces, on which the remaining provinces rely for referring patients with NETs. However, there is currently no complete explanation for the higher prevalence of NETs in these two provinces.

Although the two cohorts differed in size and date of investigation, each group displayed similarities in the mean age and age range of the patients diagnosed with NETs. The findings of the present study revealed that the majority of NETs were diagnosed within the study population with a mean age of 60 years, and almost all cases were diagnosed within the 40-80 years age group. In contrast to these findings, previous studies revealed that the median age for diagnosis of NETs in Chile was 53 years and in Argentina 53.2 years, which are slightly lower compared with the age in the present study (8,9). The differences in the incidence of NETs by sex in the present study were not statistically significant; however, there was a higher prevalence in women in cohort 2, whereas the same frequency was observed between the two sexes in cohort 1. The majority of cases did not present with metastasis and the tumors were graded as G1. These results are consistent with the findings of previous studies and may be explained by the increase in early diagnosis and improved diagnostic tools (16,17).

Classifying and staging NETs requires multiple invasive or non-invasive procedures, imaging techniques, immunohistochemistry and the detection of biomarkers such as SYN, Ki-67 and CgA (18-20). In the current study, the most frequent procedure for determining the characteristics of the NETs in both groups was a CT scan with contrast, followed by several invasive procedures, including colonoscopies, endoscopies and ultrasounds. CT scans with contrast are readily available in the participating hospitals, CHM, HST and ION, but access to this diagnostic method is limited in the other provinces of Panama. It is important to mention that other functional imaging tools, such as positron emission tomography and octreotide scans, are currently unavailable in the Republic of Panama. Confirmation of diagnosis was performed using histopathological analysis.

The identification of established diagnostic and prognostic markers of NETs in the present cases was performed at the ION. SYN is considered as the most sensitive biomarker, whereas CgA is the most specific for NETs (21,22). In addition, the percentage of Ki-67 staining is often used as a proliferation marker (23,24). Test yields were calculated for each of the immunohistology markers detected; the results revealed that, on average, SYN had the highest yield (67.0%), followed by CgA (54.8%) and CD56 (19.1%). These results are consistent with previous studies reporting that CD56 has the lowest yield (25).

The data of the present study revealed that the most frequent primary sites of NETs were the colorectum, pancreas and stomach. It has been reported that the location of the primary site varies significantly in different countries, which may be associated with undetermined factors (2). For example, the most frequent primary sites for NETs in a study involving patients from Taiwan are the rectum, lungs and stomach (26). By contrast, data from Latin American countries, such as Argentina, report that the small intestine, pancreas and colorectum are the three most frequent sites (8). In the USA, particularly in the state of Kentucky, which has a population size similar to Panama, the most frequent primary tumor site is the lung, followed by the small intestine and colorectum (2). These observed differences among countries support the requirement for further investigations to identify the factors underlying the anatomical location of NETs.

Similar to other populations worldwide, the most frequent organ affected by the metastasis of NETs is the liver (27,28), accounting for 33.8% of all cases in the present study. Other metastatic organs were also reported; however, all other sites had a frequency of <10%. In addition, the presence of metastasis was not reported or remained undetermined in 54.1% of all cases. This may be due to a combination of factors, such as the lack of early diagnosis, technological limitations in determining the presence of small lesions, or the lack of available data from clinical records.

Survival analysis for the present cohorts were adjusted for age. Following the determination of OS by age group, elderly patients (>80 years old) had a worse prognosis, with a median survival of 68.8 weeks compared with 139.4 weeks for the 40-60 years age group. These results indicated that age may contribute to worse prognosis, consistent with previous findings (29). Other confounding factors were not included in the present analysis. By contrast, analysis of the OS according to sex revealed no statistically significant difference between the two sexes. Following the determination of OS by anatomical location of the primary tumor, the data suggested that patients with a primary lesion located in the appendix or stomach had a longer OS, whereas the OS of patients with primary tumors located in the jejunum and pancreas was shorter. By contrast, patients with lung and colorectal tumors exhibited a longer OS. These data are consistent with other previous studies reporting that the shortest OS was observed among patients with primary pancreatic and intestinal cancers (4,16,26).

There were several limitations to the present study, which are related to the current legislation that states that it is not mandatory for patients with NETs to be referred to the ION; therefore, not all NET cases may have been registered. Moreover, since cases originated from different geographical locations using a wide range of diagnostic resources, the identification of cases is more likely to occur in the main provinces with more complex, developed healthcare infrastructures. Finally, during the process of data collection and analysis, the World Health Organization 2010 classification (10) for NET tumors was used instead of the 2019 guidelines (29), which may also limit our ability to compare our data to recent publications that use the 2019 classification. This is due to the implementation of the new guidelines after data collection for the present study had been completed.

In conclusion, the incidence of NETs has been reported to be increasing in the Republic of Panama. From an epidemiological perspective, the present study found that NETs behave in a similar manner compared with reports from other countries. As the first nationwide NET database, the present study aimed to further understand the characteristics of NETs in the Republic of Panama, and provided important epidemiological findings from patients and further OS data. These results may benefit the Republic of Panama by providing evidence to peers and healthcare authorities in order to allocate more financial resources to obtain additional equipment and training to enable the early diagnosis of NETs. The present study also highlights the strengths and weaknesses of the healthcare system in the Republic of Panama, and it is evident that the coordination between provinces in referring NET cases should be improved throughout the country.

## Supplementary Material

Primary tumor site and grading.

## Figures and Tables

**Table I:** Combined characteristics of all 157 patients in the present study.

Characteristics	Value, n	Value, %	95% CI
Mean age, years	60		-
Age range, years	20-93		-
Sex			
Male	65	41.40	33.61-49.53
Female	92	58.60	50.47-66.39
Age, years			
<40	16	10.19	5.94-16.02
40-59	58	36.94	29.39-45.00
60-80	73	46.50	38.51-54.62
>80	10	6.37	3.10-11.40
Metastasis			
Yes	72	45.86	37.89-53.99
No	85	54.14	46.01-62.11
Tumor grade			
G1	73	46.50	38.51-54.62
G2	31	19.75	13.83-26.84
G3	45	28.66	21.74-36.41
Unspecified	8	5.10	2.23-9.79

**Table II:** Frequency of diagnostic procedures for patients with neuroendocrine tumors.

Procedures	Value, n	Value, %	95% CI
CT with contrast	76	59.38	50.34-67.96
Colonoscopy	17	13.28	7.93-20.41
Endoscopy	14	10.94	6.11-17.67
Ultrasound	12	9.38	4.94-15.8
Mammography	2	1.56	0.19-5.53
Radiography	2	1.56	0.19-5.53
Magnetic resonance imaging	2	1.56	0.19-5.53
Bone scan	1	0.78	0.02-4.28
Cystoscopy	1	0.78	0.02-4.28
Colposcopy	1	0.78	0.02-4.28
Total	128^a^	100.00	-

^a^For all 157 patients, a total of 128 diagnostic procedures were documented. Cases with unavailable data were not included.

**Table III:** Markers used to diagnose and classify neuroendocrine tumor cases ordered by test yield.

Markers	Value, n	Test yield, %
Synaptophysin	109	67.52
Chromogranin A	104	55.41
Ki-67	136	52.20
CD56	31	19.11

**Table IV:** Frequency of primary neuroendocrine tumor location.

Sites	Value, n	Value, %	95% CI
Colorectum	27	17.20	11.65-24.03
Pancreas	20	12.74	7.96-18.99
Stomach	19	12.10	7.45-18.25
Jejunum-ileum	14	8.92	4.96-14.51
Lungs	12	7.64	4.01-12.97
Appendix	11	7.01	3.55-12.19
Breast	9	5.73	2.65-10.60
Duodenum	5	3.18	1.04-7.28
Ovaries	4	2.55	0.70-6.39
Skin	3	1.91	0.40-5.48
Others	15	9.55	5.45-15.27
Undetermined	18	7.01	6.94-17.51
Total	157	100.00	-

**Table V:** Frequency of metastasis and metastatic sites.

Sites	Value, n	Value, %	95% CI
Liver	53	33.76	26.41-41.73
Liver only	36	22.93	-
Liver and peritoneum	7	4.46	-
Liver and lungs	4	2.55	-
Others	6	3.82	-
Brain	4	2.55	0.70-6.39
Bone	2	1.27	0.15-4.53
Peritoneum	2	1.27	0.15-4.53
Other sites	11	7.01	3.55-12.19
Undetermined^a^	85	54.14	46.01-62.11
Total	157	100.00	-

^a^Cases with no identified metastasis.

## Data Availability

The datasets used and/or analyzed during the current study are available from the corresponding author on reasonable request.
