# Supplementary material for: Neuroendocrine tumors in Panama: A nationwide database analysis
Source: Mol Clin Oncol. 2021 Jun 11;15(2):157. doi: 10.3892/mco.2021.2319 (PMC8220651; doi:10.3892/mco.2021.2319)
Supplement: Primary tumor site and grading. [file Supplementary_Data.pdf]

Table SI. Primary tumor site and grading.

Anatomical site	n	%	G1	G2	G3	Grading not specified
Colorectal	27	17.20	8	8	11	0
Pancreas	20	12.74	11	7	1	1
Stomach	19	12.10	10	2	7	0
Jejunum-Ileum	14	8.92	12	1	0	1
Lungs	12	7.64	4	0	5	3
Appendix	11	7.01	7	1	3	0
Unknown primary site	18	11.46	4	4	7	1
Breast	9	5.73	6	2	0	1
Duodenum	5	3.18	1	2	2	0
Ovaries	4	2.55	2	2	0	0
Skin	3	1.91	0	0	3	0
Others	15	9.55	6	2	6	1
Total	157	100	71	31	45	8

Cases were the primary tumor site were not determined or not identified were grouped as unknown. n: Occurrence; G1, G2, G3: Tumor grading.
